# Significance of Multi-Cancer Genome Profiling Testing for Breast Cancer: A Retrospective Analysis of 3326 Cases from Japan’s National Database

**DOI:** 10.3390/genes15060792

**Published:** 2024-06-17

**Authors:** Kyoka Kawabata, Hinano Nishikubo, Saki Kanei, Rika Aoyama, Yuki Tsukada, Tomoya Sano, Daiki Imanishi, Takashi Sakuma, Koji Maruo, Yurie Yamamoto, Qiang Wang, Zhonglin Zhu, Canfeng Fan, Masakazu Yashiro

**Affiliations:** 1 Department of Molecular Oncology and Therapeutics, Osaka Metropolitan University Graduate School of Medicine, 1-4-3 Asahi-machi, Abeno-ku, Osaka 545-8585, Japan; sn23131y@st.omu.ac.jp (K.K.); sn23089k@st.omu.ac.jp (H.N.); sn23107b@st.omu.ac.jp (S.K.); si22406x@st.omu.ac.jp (R.A.); sr24464c@st.omu.ac.jp (Y.T.); sb24524y@st.omu.ac.jp (T.S.); stuck.on.alizee@gmail.com (D.I.); so22500y@st.omu.ac.jp (T.S.); k.maruo0720@gmail.com (K.M.); yurieyamamoto917@gmail.com (Y.Y.); qiangw@vip.126.com (Q.W.); zhuzhonglin1992@163.com (Z.Z.); fancanfeng@gmail.com (C.F.); 2 Cancer Center for Translational Research, Osaka Metropolitan University Graduate School of Medicine, 1-4-3 Asahi-machi, Abeno-ku, Osaka 545-8585, Japan

**Keywords:** breast cancer, multi-cancer genome profiling, Center for Cancer Genomics and Advanced Therapeutics

## Abstract

**Background:** Breast cancer (BC) has the highest morbidity rate and the second-highest mortality rate of all cancers among women. Recently, multi-cancer genome profiling (multi-CGP) tests have become clinically available. In this study, we aimed to clarify the significance of multi-CGP testing of BC by using the large clinical dataset from The Center for Cancer Genomics and Advanced Therapeutics (C-CAT) profiling database in Japan. **Materials and Methods:** A total of 3744 BC cases were extracted from the C-CAT database, which enrolled 60,250 patients between June 2019 and October 2023. Of the 3744 BC cases, a total of 3326 cases for which the C-CAT included information on ER, PR, and HER2 status were classified into four subtypes, including TNBC, HR+/HER2−, HR+/HER2+, and HR−/HER2+. Comparisons between groups were performed by the χ^2^ test or Fisher’s exact test using EZR. Kaplan–Meier curves were created using the log-rank test. **Results**: Of all 3326 cases analyzed, 1114 (33.5%) were TNBC cases, HR+/HER2− accounted for 1787 cases (53.7%), HR+/HER2+ for 260 cases (7.8%), and HR−/HER2+ for 165 cases (5.0%). Genetic abnormalities were most frequently detected in *TP53* (58.0%), *PIK3CA* (35.5%), *MYC* (18.7%), *FGF19* (15.5%), and *GATA3* (15.1%) across all BCs. The rate of TMB-High was 12.3%, and the rate of MSI-High was 0.3%, in all BC cases. Therapeutic drugs were proposed for patients with mutations in six genes: *PIK3CA*, *ERBB2*, *PTEN*, *FGFR1*, *ESR1*, and *AKT1*. The prognoses of HR+/HER2− cases were significantly (*p* = 0.044) better in the treated group than in the untreated group. **Conclusions**: These findings suggest that cancer gene panel testing is useful for HR+/HER2− cases.

## 1. Introduction

Among women, breast cancer (BC) has the highest morbidity rate and the second-highest mortality rate of all cancers [1]. BCs were recently classified into four types based on the expression of hormone receptor (HR), including estrogen receptor (ER) and progesterone receptor (PR), as well as human epidermal growth factor receptor type 2 (HER2) [2]. Triple-negative breast cancer (TNBC) is defined as a BC type that expresses neither HR nor HER2. TNBC has mainly been treated with chemotherapy, but poly ADP-ribose polymerase (PARP) inhibitors and immune checkpoint inhibitors are now also being used [3]. In contrast, hormone receptor-positive and/or HER2-positive breast cancer types are effectively treated with hormonal therapy or Herceptin therapy. The high morbidity and mortality of BC highlight the need for the development of companion diagnostic methods and new therapeutic drugs.

Recently, multi-cancer genome profiling (multi-CGP) tests have become clinically available for patients with advanced-stage solid cancers. In multi-CGP testing, therapeutic drugs are selected for patients based on comprehensive analysis of cancer-related genetic abnormalities, a practice known as precision medicine. Between 10% and 20% of all cancer cases worldwide have been reported to involve patients who were administered new drugs based on multi-CGP testing. In Japan, multi-CGP testing has become available for refractory BC patients who have either completed or nearly completed standard treatments. The Center for Cancer Genomics and Advanced Therapeutics (C-CAT) has accumulated genomic and clinical information on cancer patients who have undergone multi-CGP tests since June 2019 [4,5]. Genomic data and clinical information of all patients who have provided informed consent have been aggregated at the C-CAT of the Ministry of Health, Labour and Welfare in Japan and are being used for clinical research and drug discovery. The C-CAT Research-Use Portal site is available for research purposes with permission from the C-CAT, and around sixty thousand patients with a malignant solid tumor have been included in the current database so far (https://www.ncc.go.jp/en/c_cat/use/index.html (accessed on 10 June 2024)). Currently, it has been reported that the most common tumor type is Bowel, followed by Pancreas, Biliary Tract, and Breast, for all CGP tests in Japan [6]. In this study, we aimed to clarify the significance of multi-CGP testing for BC by leveraging the extensive clinical data from the C-CAT profiling database in Japan. 

## 2. Materials and Methods

### 2.1. BC Patients and Subtype Classification

**Figure 1:**
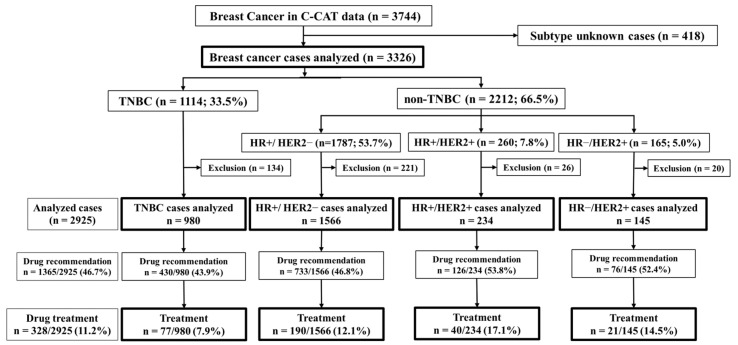
Patient background. The number of excluded cases and the number of cases used for analysis are shown. Also shown is the number of cases by subtype (TNBC, HR+/HER2−, HR+/HER2+, HR−/HER2+), treatment attainment rate, and treatment achievement rate. HR, hormone receptor; HER2, human epidermal growth factor receptor type 2; TNBC, triple-negative breast cancer.

A total of 3744 BC cases were extracted from the C-CAT database, which enrolled 60,250 patients between June 2019 and October 2023. Of the 3744 BC cases, 418 were excluded from the analysis because of a lack of information about HR and HER2 status. A total of 3326 cases were classified into four subtypes according to C-CAT information on ER, PR, and HER2 status (Figure 1). Based on the expression of ER, PR, and HER2, TNBC was defined as HR−/HER2−. A total of 134 TNBC cases, 221 HR+/HER2− cases, 26 HR+/HER2+ cases, and 20 HR−/HER2+ cases were excluded from the analysis of treatment attainment rates and treatment achievement rates because of a lack of clinicopathologic information.

### 2.2. Multi-CGP Testing

Patients were examined by 4 types of CGP tests, namely the OncoGuide™ NCC Oncopanel System (NCC) (Sysmex Co., Ltd., Kobe, Japan), FoundationOne^®^ CDx (F1CDx) (Foundation Medicine Inc., Cambridge, MA, USA), FoundationOne^®^ Liquid CDx (F1L) (Foundation Medicine Inc.), and Guardant360^®^ CDx sequencing technology (Guardant) (Guardant Health, Palo Alto, CA, USA). NCC analysis was performed using DNA from tumor tissue samples and circulating tumor DNA from blood, F1CDx analysis was performed using genomic DNA extracted from tumor tissue samples, F1L analysis was performed using cell-free DNA extracted from patient plasma, and Guardant analysis was performed using cell-free DNA extracted from whole blood samples. NCC analysis was performed for 361 cases, F1CDx for 2420 cases, F1L for 539 cases, and Guardant for 6 cases.

### 2.3. Clinical and Genomic Database and Indication for Multi-CGP Tests

A retrospective study was conducted using the C-CAT database, which includes clinicopathologic information such as age, gender, Eastern Cooperative Oncology Group Performance Status (ECOG-PS) [7], cancer type, pathological findings, chemotherapy regimen, and best response, as well as genomic information such as mutation types, allele frequencies, rearrangements, microsatellite instability (MSI), and tumor mutation burden (TMB). Multi-CGP tests were recommended for cancer patients who had completed their standard treatment or for those who had not yet received standard treatment.

### 2.4. Genetic Abnormalities and Therapeutic Efficacy

The NCC test is designed to examine mutations, amplifications, and homozygous deletions of the entire coding region of 114 genes of clinical or preclinical relevance, along with rearrangements of 13 oncogenes, MSI, and TMB. F1CDx and F1L detect substitutions, insertions, deletions, and copy number alterations in 324 genes and selective gene rearrangements and genomic signatures including MSI and TMB. Guardant detects single-nucleotide variants, indels, fusions, and copy number alterations in 74 genes, and MSI. To evaluate truly targetable genomic mutations, only variants that were assessed as “oncogenic”, “pathogenic”, “likely oncogenic”, and “likely pathogenic” in the clinical annotation of C-CAT findings were extracted, and variants of unknown significance (VUS) were not included. The clinical annotation in C-CAT is based on the Cancer Knowledge Data Base (CKDB), constructed by C-CAT, which aggregates data on gene mutations, drugs, and clinical trials from various public genomic medicine-related databases available worldwide. Therapeutic efficacy was determined by the best response efficacy, as follows: complete response (CR), partial response (PR), stable disease (SD), progressive disease (PD), and not evaluable (NE). Disease control rate (DCR) was determined for each subtype. DCR was defined as the proportion of all enrolled patients showing CR, PR, or SD. In this study, we investigated overall survival (OS), calculated as the duration from the point of registry in the C-CAT database to death from any cause. Due to the nature of the database, which is based on data manually entered by each attending physician, some cases were found to have missing data. 

### 2.5. Statistical Analysis

Group comparisons were conducted using the χ^2^ test or Fisher’s exact test with Bonferroni correction. OS was calculated using the Kaplan–Meier method. Log-rank tests were used to determine significant differences between the two groups. All statistical tests were performed using a significance cutoff of *p* < 0.05. Statistical analyses were conducted using the EZR (Easy R) software package version 1.65 (Saitama Medical Center, Jichi Medical University, Saitama, Japan) and SPSS^®^ version 28 (IBM Corp., Armonk, NY, USA).

## 3. Results

### 3.1. Subtype and Drug Treatment

Figure 1 shows the number of patients used for this analysis. Of all 3326 cases analyzed, 1114 (33.5%) were TNBC cases, and 2212 (66.5%) were non-TNBC cases. Among the 2212 non-TNBC cases, HR+/HER2− accounted for 1787 cases (53.7%), HR+/HER2+ for 260 cases (7.8%), and HR−/HER2+ for 165 cases (5.0%). The drug recommendation rate and medication rate were highest in HR+/HER2+ cases (53.8% and 17.1%), followed in order by HR−/HER2+ cases (52.4% and 14.5%), HR+/HER2− cases (46.8% and 12.1%), and TNBC cases (43.9% and 7.9%). Table 1 summarizes the clinical characteristics for each subtype: age, ECOG-PS, materials, smoking, and alcohol. TNBC cases were significantly (*p* < 0.001) younger compared to HR+/HER2− cases. In contrast, no significant differences were noted among the 4 groups in terms of ECOG-PS, smoking, and alcohol. Tissue samples were used for 1022 (91.7%) cases of TNBC, 1400 (78.3%) cases of HR+/HER2−, 217 (83.5%) cases of HR+/HER2+, and 142 (86.1%) cases of HR−/HER2+. Tissue samples were significantly (*p* < 0.001) frequent for TNBC compared to HR+/HER2− cases and HR+/HER2+ cases.

### 3.2. Genomic Abnormalities

**Figure 2:**
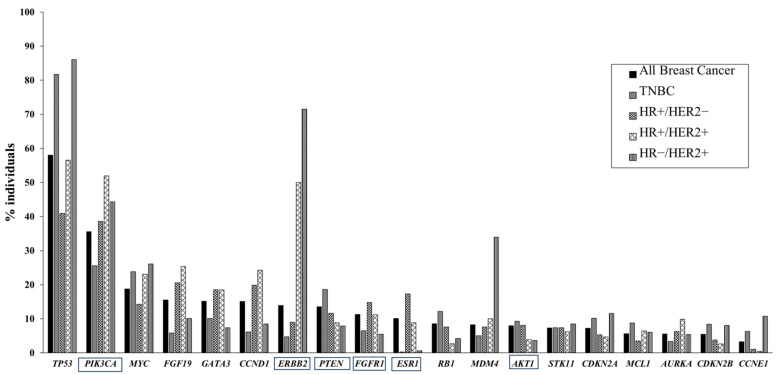
Genomic abnormalities. The top genes with abnormalities in BC. The proportions of genes with abnormalities in BC are shown. Proportions for all BCs and subtypes are shown. Genes associated with therapeutic drug presentation are also surrounded with a square. HR, hormone receptor; HER2, human epidermal growth factor receptor type 2; TNBC, triple-negative breast cancer.

Genomic abnormalities were evaluated as pathogenic, likely pathogenic, oncogenic, or likely oncogenic. The 19 most frequently mutated genes are summarized in Figure 2. Genetic abnormalities were most frequently detected in *TP53* (58.0%), *PIK3CA* (35.5%), *MYC* (18.7%), *FGF19* (15.5%), and *GATA3* (15.1%) across all BCs. In TNBC cases, genetic abnormalities were frequently detected in *TP53* (81.7%), *PIK3CA* (25.6%), *MYC* (23.8%), *PTEN* (18.6%), and *RB1* (12.1%). In contrast, no significant differences in genomic abnormalities were observed between BC patients < 40 years and ≥ 40 years. Therapeutic drugs were proposed for six genes: *PIK3CA*, *ERBB2*, *PTEN*, *FGFR1*, *ESR1*, and *AKT1*. Abnormalities in *PIK3CA* were most frequently detected in HR+/HER2+ cases (51.9%), *ERBB2* in HR−/HER2+ cases (71.5%), *PTEN* in TNBC cases (18.6%), *FGFR1* in HR+/HER2− cases (14.8%), and *ESR1* in HR+/HER2− cases (17.3%).

Genomic abnormalities in *BRCA1* and *BRCA2* were found in 3.2% and 6.9% of all BCs. *BRCA1* abnormality was detected in 6.1%, 1.9%, 1.2%, and 1.2% of TNBC cases, HR+/HER2−, HR+/HER2+, and HR−/HER2+ cases, respectively. *BRCA2* abnormality was detected in 3.8%, 8.8%, 8.8%, and 4.2% of TNBC cases, HR+/HER2−, HR+/HER2+, and HR−/HER2+ cases, respectively. The proportion of MSI-High cases was 0.3% (*n* = 10/3100) across all BC cases, including 0.5% (*n* = 5/1034) in TNBC and 0.3% (*n* = 5/1667) in HR+/HER2−, while there was no MSI-High among HER2+ cases. TMB-High, defined as TMB ≥ 10 mut/Mb, was 12.3% (*n* = 407/3320) in all BC cases, including 8.5% (*n* = 95/1114) in TNBC, 14.1% (*n* = 251/1781) in HR+/HER2−, 12.3% (*n* = 32/260) in HR+/HER2+, and 17.6% (*n* = 29/165) in HR−/HER2+. There were 49 cases with TMB ≥ 10 mut/Mb who were confirmed to have received Pembrolizumab.

### 3.3. Survival

**Figure 3:**
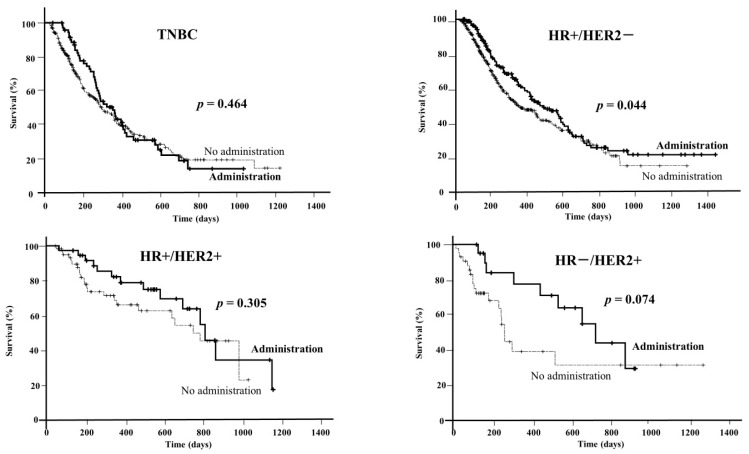
Survival curves for groups with and without therapeutic drug administration. Comparison of prognosis results between cases in which the proposed therapeutic drugs were not administered and cases in which they were administered, by subtype. Of the cases for which therapeutic drugs were presented, the date of confirmation of survival and date of death were noted (*n* = 1121). There were 352 cases of TNBC and 769 cases of non-TNBC (HR+/HER2−: 608 cases, HR+/HER2+: 98 cases, HR−/HER2+: 63 cases). Of these, 73 cases of TNBC and 236 cases of non-TNBC (HR+/HER2−: 178 cases, HR+/HER2+: 37 cases, HR−/HER2+: 21 cases) were treated with the proposed therapeutic drug. The thick line indicates the administered group, and the dotted line indicates the non-administered group. HR, hormone receptor; HER2, human epidermal growth factor receptor type 2; TNBC, triple-negative breast cancer.

Prognoses were compared between patients treated by the proposed therapy and those without such treatment (Figure 3). Among TNBC cases, there was no significant prognostic difference between these groups (*p* = 0.464). On the other hand, in HR+/HER2− cases, the median OSs in the administration and non-administration groups were 468 days and 343 days, respectively. The prognoses of HR+/HER2− cases were significantly (*p* = 0.044) better in the treated group, whereas among HR+/HER2+ and HR−/HER2+ cases there was no significant difference between the treated and untreated groups.

### 3.4. Therapeutic Response by Proposed Drugs

Table 2 summarizes the treatment line prior to the proposed therapies. Most patients had received standard treatment before the administration of the proposed drugs. There were no significant differences in treatment policy among the four subtypes. The fifth and subsequent treatment lines were administered for TNBC, HR+/HER2−, HR+/HER2+, and HR−/HER2+ in 40 cases (51.9%), 150 cases (78.9%), 31 cases (77.5%), and 16 cases (76.2%), respectively. The third or fourth treatment lines were administered for TNBC, HR+/HER2−, HR+/HER2+, and HR−/HER2+ in 26 cases (33.8%), 15 cases (7.9%), 4 cases (10.0%), and 3 cases (14.3%). TNBC cases were more frequently administered third- or fourth-line treatment compared to the other subtypes. The best response judgments after administration are summarized in Table 2. Among the 328 administration cases, responders (defined as having achieved CR, PR, or SD) were observed in 19 cases (24.7%) of TNBC, 71 cases (37.4%) of HR+/HER2−, 19 cases (47.5%) of HR+/HER2+, and 14 cases (66.7%) of HR−/HER2+. Responders were high in HR+/HER2+ cases and HR−/HER2+ cases. TNBC cases exhibited significantly lower disease control rates compared to HR−/HER2+ cases (*p* < 0.01).

## 4. Discussion

We conducted a retrospective study of patients with BC based on CGP in Japan from the nationwide C-CAT database. This study reported the significance of multi-CGP testing for BC patients based on a large cohort, including 3326 cases in Japan. The prevalence of multi-CGP testing cases was relatively high (33.5%) in TNBC, whereas TNBC typically accounts for 15–20% of all BCs [8]. In contrast, the druggable rate and drug treatment rate were significantly lower in TNBC cases compared to non-TNBC cases. Promising driver genes for TNBC might not be included in the current four types of multi-CGP testing, suggesting that multi-CGP testing might not be useful for TNBC patients in terms of drug administration.

Pathogenic mutations were frequently found in *TP53*, *PIK3CA*, *MYC*, *FGF19*, and *GATA3* in the 3326 BC cases. Among genes associated with therapeutic drug presentation, abnormalities in *PIK3CA*, *ERBB2*, and *PTEN* were frequently found among the 3326 BC cases. Abnormalities in *PTEN* and *AKT1* were frequently found in TNBC cases. In contrast, abnormalities in *ESR1* and *FGFR1* were frequent in HR+/HER2− cases, abnormalities in *PIK3CA* were frequent in HR+/HER2+ cases, and abnormalities in *ERBB2* were common in HR−/HER2+ cases. Everolimus [9] and Capivasertib [10] are indicated for *PTEN*/*PIK3CA*/*AKT1* alterations, alterations in *ESR1* can be treated with hormonal therapy [11], and Herceptin can be used as an anti-HER2 drug [12]. Currently, therapeutic drugs for abnormalities in *FGFR1* are in the clinical trial stage [13], and approval of clinical drugs for abnormalities in *FGFR1* might be urgently expected for BC.

It has been reported that abnormalities in several genes such as *TOP2A*, *CCNA2*, *PCNA*, *MSH2*, *CDK6*, *DGKH*, *GADD45B*, *KLF7*, *LYST*, *NR6A1*, *PYCARD*, *ROBO1*, *SLC22A20P*, *SLC24A3*, and *SLC45A4* are useful as markers for the prognosis of TNBC and provide potential therapeutic targets [14,15]. In this study, *CDK6* and *MSH2* were included in the multi-CGP tests, and *CDK6* and *MSH2* alterations were found in 2.2% and 0.4% of all TNBCs, respectively. *CDK6* amplification is considered an indication for treatment with drugs such as abemaciclib, palbociclib, and ribociclib in Japan [16]. These findings suggest that *CDK6* abnormality might be a useful marker for potential therapeutic targets for TNBC, while the frequency of *CDK6* amplification was low in TNBC. Although epithelial–mesenchymal transition (EMT) gene signatures, such as Slug, Snail, Transforming growth factor (TGF)-β, and N-cadherin, have been reported to be useful markers for the prognosis of TNBC [17,18,19], these EMT-associated genes were not included in the multi-CGP tests.

Genomic abnormalities of *BRCA1* and *BRCA2* were found in 3.2% and 6.9% of all BCs. *BRCA1* abnormality was highly detected in TNBC cases, while *BRCA2* abnormality was highly detected in HR+ cases known as Luminal types, which resembles some previous reports [20]. *BRCA1* and *BRCA2* were the most common genes with abnormalities, followed by presumed germline pathogenic variants (PGPVs). *BRCA1* and *BRCA2* with PGPVs show high frequency in HBOC [21]. It has been reported that hereditary breast and ovarian cancer (HBOC), caused by a germline pathogenic variant of *BRCA1* or *BRCA2*, is one of the common hereditary tumors [22], suggesting that around 5% of patients with BC might show HBOC in Japan.

MSI-High is a companion diagnostic biomarker predicting the response to immune checkpoint inhibitors (ICIs) [23]. MSI-Low is reported to account for about 3% of BCs [24], and MSI-High and MSI-Low cases were found to be infrequent in BC. The frequency of MSI-High in BC is reported to be low, at less than 1% [25]. In this study also, only 0.5% of TNBC cases and 0.3% of HR+/HER2− cases were MSI-High. These findings might suggest that MSI-High alone is not sufficient for a companion diagnostic test to determine the indication of ICIs for BC. In contrast, about 12% of BC patients showed TMB-High as a companion diagnostic test for ICIs. There was a significant difference in frequency between MSI-High and TMB-High. It has been reported that patients with TMB ≥ 14 mut/mb have a higher response rate than patients with TMB ≥ 9 and < 14 mut/Mb [26]. Since the overall response rate of ICIs for BC is reported to be 7.1% [27,28], setting the TMB-High cutoff value above 10 mut/Mb might be more appropriate. It would be necessary to determine a new cutoff value for TMB-High as a companion marker for the application criteria for ICIs.

A report of multi-CGP tests using liquid biopsy with circulating tumor DNA (ctDNA) indicated that *TP53* and/or *BRCA1* mutations could be useful prognostic markers in TNBC patients in Japan [29]. Multi-CGP tests for TNBC patients might be useful for analyses using ctDNA from liquid biopsies, while only 8.3% of all TNBCs were analyzed using liquid biopsy in this study (Table 1). A report of multi-CGP tests from China indicated that *RB1* mutation was found in 30% of cases and was associated with shorter disease-free survival (DFS) in patients with TNBC in the Chinese population [30], but our data did not show shorter DFS. Since the *RB1* mutation in TNBC exhibited a low frequency of 12.1% in this study, *RB1* mutation might not be associated with DFS in patients with TNBC in the Japanese population. The difference in the frequency of *RB1* mutation might be due to ethnic differences between Chinese and Japanese patients.

There was no significant difference in prognosis between patients with TNBC and those without it. On the other hand, in non-TNBC cases, prognosis was significantly better with treatment than without treatment. These findings suggest that cancer gene panel testing is useful for non-TNBC cases. In particular, a significant difference was observed in HR+/HER2− cases. These findings suggest that multi-CGP tests are useful for HR+/HER2− cases in which standard treatment has been completed.

It has been reported that some clinical features such as obesity and diabetes are associated with poor prognosis in TNBC [31,32]; however, the C-CAT database did not include these clinical features. These clinical factors might be necessary to analyze the prognosis of TNBC.

There are limitations in this study. While the C-CAT database provided comprehensive results of genomic and clinicopathologic information, some information, such as current progression-free survival and hereditary tumors, is not available. This study lacks data from patients without medical insurance due to the universal health insurance system in Japan.

In conclusion, multi-CGP testing might be useful for non-TNBC cases, especially for HR+/HER2− cases.

## Figures and Tables

**Table 1:** Patients’ clinicopathologic characteristics.

All Cases (*n* = 3326)	TNBC (*n* = 1114)	HR+/HER2− (*n* = 1787)	HR+/HER2+ (*n* = 260)	HR−/HER2+ (*n* = 165)	*p*-Value
Age					<0.001
<40 (*n* = 258)	*n* = 131 (11.8%)	*n* = 94 (5.3%)	*n* = 21 (8.1%)	*n* = 12 (7.3%)	
≥40 (*n* = 3068)	*n* = 983 (88.2%)	*n* = 1693 (94.7%)	*n* = 239 (91.9%)	*n* = 153 (92.7%)	
ECOG-PS					0.347
PS0, PS1 (*n* = 3192)	*n* = 1077 (96.7%)	*n* = 1707 (95.5%)	*n* = 250 (96.2%)	*n* = 158 (95.8%)	
PS2, PS3, PS4 (*n* = 102)	*n* = 25 (2.2%)	*n* = 61 (3.4%)	*n* = 9 (3.5%)	*n* = 7 (4.2%)	
Unknown (*n* = 32)	*n* = 12 (1.1%)	*n* = 19 (1.1%)	*n* = 1 (0.4%)	*n* = 0 (0%)	
Materials					<0.001
Tissue (*n* = 2781)	*n* = 1022 (91.7%)	*n* = 1400 (78.3%)	*n* = 217 (83.5%)	*n* = 142 (86.1%)	
Blood (*n* = 545)	*n* = 92 (8.3%)	*n* = 387 (21.7%)	*n* = 43 (16.5%)	*n* = 23 (13.9%)	
Smoking					0.235
Smoking (*n* = 627)	*n* = 223 (20.0%)	*n* = 311 (17.4%)	*n* = 62 (23.8%)	*n* = 31 (18.8%)	
No Smoking (*n* = 2548)	*n* = 844 (75.8%)	*n* = 1391 (77.8%)	*n* = 186 (71.5%)	*n* = 127 (77.0%)	
Unknown (*n* = 151)	*n* = 47 (4.2%)	*n* = 85 (4.8%)	*n* = 12 (4.6%)	*n* = 7 (4.2%)	
Alcohol					0.513
Heavy alcohol (*n* = 150)	*n* = 47 (4.2%)	*n* = 83 (4.6%)	*n* = 12 (4.6%)	*n* = 8 (4.8%)	
No heavy alcohol (*n* = 2862)	*n* = 972 (87.3%)	*n* = 1519 (85.0%)	*n* = 230 (88.5%)	*n* = 141 (85.5%)	
Unknown (*n* = 314)	*n* = 95 (8.5%)	*n* = 185 (10.4%)	*n* = 18 (6.9%)	*n* = 16 (9.7%)	

TNBC, triple-negative breast cancer; HR, hormone receptor; HER2, human epidermal growth factor receptor type 2; ECOG-PS (Eastern Cooperative Oncology Group-Performance Status) [6]. PS0, fully active, able to carry out all pre-disease activities without restriction; PS1, restricted in physically strenuous activity but ambulatory and able to carry out work of a light or sedentary nature, e.g., light house work, office work; PS2, ambulatory and capable of all self-care but unable to carry out any work activities, up and about more than 50% of waking hours; PS3, capable of only limited self-care, uses bed or chair more than 50% of waking hours; PS4, completely disabled, cannot carry out any self-care, or only uses bed or chair.

**Table 2:** Treatment policy and line for cases that received treatment and therapeutic response to the proposed drugs.

Drug Treatment (*n* = 328)	TNBC (*n* = 77)	HR+/HER2− (*n* = 190)	HR+/HER2+ (*n* = 40)	HR−/HER2+ (*n* = 21)	*p*-Value
Treatment policy					
Insurance medical treatment (*n* = 283)	*n* = 61 (79.2%)	*n* = 165 (86.8%)	*n* = 38 (95.0%)	*n* = 19 (90.5%)	
Corporate clinical trial (*n* = 29)	*n* = 8 (10.4%)	*n* = 17 (8.9%)	*n* = 2 (5.0%)	*n* = 2 (9.5%)	
Physician-initiated trial (*n* = 7)	*n* = 4 (5.2%)	*n* = 3 (1.6%)	*n* = 0 (0%)	*n* = 0 (0%)	
Others (*n* = 9)	*n* = 4 (5.2%)	*n* = 5 (2.6%)	*n* = 0 (0%)	*n* = 0 (0%)	
Treatment line					
1st, 2nd (*n* = 32)	*n* = 7 (9.1%)	*n* = 19 (10.0%)	*n* = 4 (10.0%)	*n* = 2 (9.5%)	
3rd, 4th (*n* = 48)	*n* = 26 (33.8%)	*n* = 15 (7.9%)	*n* = 4 (10.0%)	*n* = 3 (14.3%)	
5th≤ (*n* = 237)	*n* = 40 (51.9%)	*n* = 150 (78.9%)	*n* = 31 (77.5%)	*n* = 16 (76.2%)	
Unknown (*n* = 11)	*n* = 4 (5.2%)	*n* = 6 (3.2%)	*n* = 1 (2.5%)	*n* = 0 (0%)	
Therapeutic response					
CR (*n* = 1)	*n* = 0 (0%)	*n* = 1 (0.5%)	*n* = 0 (0%)	*n* = 0 (0%)	
PR (*n* = 48)	*n* = 9 (11.7%)	*n* = 27 (14.2%)	*n* = 6 (15.0%)	*n* = 6 (28.6%)	
SD (*n* = 74)	*n* = 10 (13.0%)	*n* = 43 (22.6%)	*n* = 13 (32.5%)	*n* = 8 (38.1%)	
PD (*n* = 79)	*n* = 24 (31.2%)	*n* = 45 (23.7%)	*n* = 7 (17.5%)	*n* = 3 (14.3%)	
NE (*n* = 126)	*n* = 34 (44.2%)	*n* = 74 (38.9%)	*n* = 14 (35.0%)	*n* = 4 (19.0%)	
Response efficacy rate					
CR, PR, SD (*n* = 123)	*n* = 19 (24.7%)	*n* = 71 (37.4%)	*n* = 19 (47.5%)	*n* = 14 (66.7%)	
PD, NE (*n* = 205)	*n* = 58 (75.3%)	*n* = 119 (62.6%)	*n* = 21 (52.5%)	*n* = 7 (33.3%)	<0.01

TNBC, triple-negative breast cancer; HR, hormone receptor; HER2, human epidermal growth factor receptor type 2; CR, complete response; PR, partial response; SD, stable disease; PD, progressive disease; NE, not evaluable.

## Data Availability

The datasets presented in this article are not readily available because the data are part of an ongoing study or due to technical limitations. Requests to access the datasets should be directed to the Center for Cancer Genomics and Advanced Therapeutics (C-CAT).

## Abbreviations

BC, breast cancer; HR, hormone receptor; ER, estrogen receptor; PR, progesterone receptor; HER2, human epidermal growth factor receptor type 2; TNBC, triple-negative breast cancer; multi-CGP, multi-cancer genome profiling; C-CAT, Center for Cancer Genomics and Advanced Therapeutics; MSI, microsatellite instability; TMB, tumor mutation burden; CR, complete response; PR, partial response; SD, stable disease; PD, progressive disease; NE, not evaluable.

## References

[1] Siegel R.L., Miller K.D., Fuchs H.E., Jemal A. (2022). Cancer statistics, 2022. CA Cancer J. Clin..

[2] Carlson R.W., Allred D.C., Anderson B.O., Burstein H.J., Carter W.B., Edge S.B., Erban J.K., Farrar W.B., Goldstein L.J., Gradishar W.J. (2009). Breast Cancer. Clinical Practice Guidelines in Oncology. J. Natl. Compr. Cancer Netw..

[3] Lebert J.M., Lester R., Powell E., Seal M., McCarthy J. (2018). Advances in the systemic treatment of triple-negative breast cancer. Curr. Oncol..

[4] Mukai Y., Ueno H. (2021). Establishment and implementation of Cancer Genomic Medicine in Japan. Cancer Sci..

[5] Kohno T., Kato M., Kohsaka S., Sudo T., Tamai I., Shiraishi Y., Okuma Y., Ogasawara D., Suzuki T., Yoshida T. (2022). C-CAT: The National Datacenter for Cancer Genomic Medicine in Japan. Cancer Discov..

[6] Yamaguchi T., Ikegami M., Aruga T., Kanemasa Y., Horiguchi S., Kawai K., Takao M., Yamada T., Ishida H. (2024). Genomic landscape of comprehensive genomic profiling in patients with malignant solid tumors in Japan. Int. J. Clin. Oncol..

[7] Oken M.M., Creech R.H., Tormey D.C., Horton J., Davis T.E., McFadden E.T., Carbone P.P. (1982). Toxicity and response criteria of the Eastern Cooperative Oncology Group. Am. J. Clin. Oncol..

[8] Metzger-Filho O., Tutt A., De Azambuja E., SSaini K., Viale G., Loi S., Bradbury I., MBliss J., AAzim Jr H., Ellis P. (2012). Dissecting the heterogeneity of triple-negative breast cancer. J. Clin. Oncol..

[9] Chen Z., Zheng Y., Cao W., Zhang Y., Zhao Z., Wang G., Zhao J., Cai S., Shao X., Huang J. (2019). Everolimus-containing therapy vs conventional therapy in the treatment of refractory breast cancer patients with PI3K/AKT/mTOR mutations: A retrospective study. Cancer Med..

[10] Browne I.M., André F., Chandarlapaty S., Carey L.A., Turner N.C. (2024). Optimal Targeting of PI3K-AKT and MTOR in Advanced Oestrogen Receptor-Positive Breast Cancer. Lancet Oncol..

[11] Damodaran S., O’Sullivan C.C., Elkhanany A., Anderson I.C., Barve M., Blau S., Cherian M.A., Peguero J.A., Goetz M.P., Plourde P.V. (2023). Open-label, phase II, multicenter study of lasofoxifene plus abemaciclib for treating women with metastatic ER+/HER2− breast cancer and an ESR1 mutation after disease progression on prior therapies: ELAINE 2. Ann. Oncol..

[12] Stanowicka-Grada M., Senkus E. (2023). Anti-HER2 Drugs for the Treatment of Advanced HER2 Positive Breast Cancer. Curr. Treat. Options Oncol..

[13] Coombes R.C., Badman P.D., Lozano-Kuehne J.P., Liu X., Macpherson I.R., Zubairi I., Baird R.D., Rosenfeld N., Garcia-Corbacho J., Cresti N. (2022). Results of the phase IIa RADICAL trial of the FGFR inhibitor AZD4547 in endocrine resistant breast cancer. Nat. Commun..

[14] Ma J., Chen C., Liu S., Ji J., Wu D., Huang P., Wei D., Fan Z., Ren L. (2022). Identification of a five genes prognosis signature for triple-negative breast cancer using multi-omics methods and bioinformatics analysis. Cancer Gene Ther..

[15] Kim C.M., Park K.H., Yu Y.S., Kim J.W., Park J.Y., Park K., Yu J.H., Lee J.E., Sim S.H., Seo B.K. (2024). A 10-Gene Signature to Predict the Prognosis of Early-Stage Triple-Negative Breast Cancer. Cancer Res. Treat..

[16] Wang R., Xu K., Gao F., Huang J., Guan X. (2021). Clinical considerations of CDK4/6 inhibitors in triple-negative breast cancer. Biochim. Biophys. Acta Rev. Cancer.

[17] Li M., Zhang L., Guan T., Huang L., Zhu Y., Wen Y., Ma X., Yang X., Wan R., Chen J. (2024). Energy stress-activated AMPK phosphorylates Snail1 and suppresses its stability and oncogenic function. Cancer Lett..

[18] Merikhian P., Eisavand M.R., Farahmand L. (2021). Triple-negative breast cancer: Understanding Wnt signaling in drug resistance. Cancer Cell Int..

[19] Zhang B., Zhao R., Wang Q., Zhang Y.J., Yang L., Yuan Z.J., Yang J., Wang Q.J., Yao L. (2023). An EMT-Related Gene Signature to Predict the Prognosis of Triple-Negative Breast Cancer. Adv. Ther..

[20] Ha S.M., Chae E.Y., Cha J.H., Kim H.H., Shin H.J., Choi W.J. (2017). Association of BRCA Mutation Types, Imaging Features, and Pathologic Findings in Patients With Breast Cancer With BRCA1 and BRCA2 Mutations. AJR Am. J. Roentgenol..

[21] Meric-Bernstam F., Brusco L., Daniels M., Wathoo C., Bailey A.M., Strong L., Shaw K., Lu K., Qi Y., Zhao H. (2016). Incidental germline variants in 1000 advanced cancers on a prospective somatic genomic profiling protocol. Ann. Oncol..

[22] Liu Y.L., Maio A., Kemel Y., Salo-Mullen E.E., Sheehan M., Tejada P.R., Trottier M., Arnold A.G., Fleischut M.H., Latham A. (2022). Disparities in cancer genetics care by race/ethnicity among pan-cancer patients with pathogenic germline variants. Cancer.

[23] Puccini A., Battaglin F., Iaia M.L., Lenz H.J., Salem M.E. (2020). Overcoming resistance to anti-PD1 and anti-PD-L1 treatment in gastrointestinal malignancies. J. Immunother. Cancer.

[24] Halford S.E.R., Sawyer E.J., Lambros M.B., Gorman P., Macdonald N.D., Talbot I.C., Foulkes W.D., Gillett C.E., Barnes D.M., Akslen L.A. (2003). MSI-low, a real phenomenon which varies in frequency among cancer types. J. Pathol..

[25] Dudley J.C., Lin M.T., Le D.T., Eshleman J.R. (2016). Microsatellite instability as a biomarker for PD-1 blockade. Clin. Cancer Res..

[26] Barroso-Sousa R., Pacífico J.P., Sammons S., Tolaney S.M. (2023). Tumor Mutational Burden in Breast Cancer: Current Evidence, Challenges, and Opportunities. Cancers.

[27] Mao Y., Xie H., Lv M., Yang Q., Shuang Z., Gao F., Li S., Zhu L., Wang W. (2023). The landscape of objective response rate of anti-PD-1/L1 monotherapy across 31 types of cancer: A system review and novel biomarker investigating. Cancer Immunol. Immunother..

[28] Arora S., Velichinskii R., Lesh R.W., Ali U., Kubiak M., Bansal P., Borghaei H., Edelman M.J., Boumber Y. (2019). Existing and Emerging Biomarkers for Immune Checkpoint Immunotherapy in Solid Tumors. Adv. Ther..

[29] Arimura A., Sakai K., Kaneshiro K., Morisaki T., Hayashi S., Mizoguchi K., Yamada M., Kai M., Ono M., Nishio K. (2024). TP53 and/or BRCA1 Mutations Based on CtDNA Analysis as Prognostic Biomarkers for Primary Triple-Negative Breast Cancer. Cancers.

[30] Wang L., Zhai Q., Lu Q., Lee K., Zheng Q., Hong R., Wang S. (2021). Clinical genomic profiling to identify actionable alterations for very early relapsed triple-negative breast cancer patients in the Chinese population. Ann. Med..

[31] Bowers L.W., Rossi E.L., McDonell S.B., Doerstling S.S., Khatib S.A., Lineberger C.G., Albright J.E., Tang X., A deGraffenried L., Hursting S.D. (2018). Leptin Signaling Mediates Obesity-Associated CSC Enrichment and EMT in Preclinical TNBC Models. Mol. Cancer Res..

[32] Song J., Du J., Han L., Lin X., Fan C., Chen G. (2023). The Effect of Metformin on Triple-Negative Breast Cancer Cells and Nude Mice. Altern. Ther. Health Med..

